# Demonstration of natriuretic activity in urine of neurosurgical patients with renal salt wasting

**DOI:** 10.12688/f1000research.2-126.v2

**Published:** 2013-06-13

**Authors:** Steven J Youmans, Miriam R Fein, Elizabeth Wirkowski, John K Maesaka

**Affiliations:** 1 Department of Biomedical Sciences, New York Institute of Technology College of Osteopathic Medicine, Old Westbury, NY, 11568, USA; 2 Graduate Program in Genetics, State University of New York, Stony Brook, NY, 21814, USA; 3 Cold Spring Harbor Laboratory, Cold Spring Harbour, NY, 11724, USA; 4 Department of Neurology, Winthrop-University Hospital, Mineola, NY, 11501, USA; 5 Department of Medicine, Division of Nephrology and Hypertension, Winthrop-University Hospital, Mineola, NY, 11501, USA; 6 SUNY Downstate Medical Center, Stony Brook, NY, 21814, USA

## Abstract

We have utilized the persistent elevation of fractional excretion (FE) of urate, > 10%, to differentiate cerebral/renal salt wasting (RSW) from the syndrome of inappropriate antidiuretic hormone secretion (SIADH), in which a normalization of FEurate occurs after correction of hyponatremia.  Previous studies suggest as well  that an elevated FEurate with normonatremia, without pre-existing hyponatremia, is also consistent with RSW, including studies demonstrating induction of RSW in rats infused with plasma from normonatremic neurosurgical and Alzheimer’s disease patients.  The present studies were designed to test whether precipitates from the urine of normonatremic neurosurgical patients, with either normal or elevated FEurate, and patients with SIADH, display natriuretic activity.

**Methods**: Ammonium sulfate precipitates from the urine of 6 RSW and 5 non-RSW Control patients were dialyzed (10 kDa cutoff) to remove the ammonium sulfate, lyophilized, and the reconstituted precipitate was tested for its effect on transcellular transport of ^22^Na across LLC-PK1 cells grown to confluency in transwells.

**Results**: Precipitates from 5 of the 6 patients with elevated FEurate and normonatremia significantly inhibited the *in vitro* transcellular transport of ^22^Na above a concentration of 3 μg protein/ml, by 10-25%, versus to vehicle alone, and by 15-40% at concentrations of 5-20 μg/ml as compared to precipitates from 4 of the 5 non-RSW patients with either normal FEurate and normonatremia (2 patients) or with SIADH (2 patients).

**Conclusion**: These studies provide further evidence that an elevated FEurate with normonatremia is highly consistent with RSW.  Evidence in the urine of natriuretic activity suggests significant renal excretion of the natriuretic factor. The potentially large source of the natriuretic factor that this could afford, coupled with small analytical sample sizes required by the *in-vitro* bioassay used here, should facilitate future experimental analysis and allow the natriuretic factor to be investigated as a potential biomarker for RSW.

## Introduction

The unresolved controversy over the prevalence of the cerebral salt-wasting, or the preferred term, renal saltwasting (RSW) syndrome, can be ascribed, in large part, to difficulties in differentiating RSW, where extracellular volume is depleted, from the syndrome of inappropriate secretion of antidiuretic hormone (SIADH), where volume is normal or expanded. The overlapping laboratory characteristics, clinical associations and our inability to assess accurately, but non-invasively and rapidly, the volume status of these patients are at the root of this diagnostic and consequential therapeutic dilemma ^1– 4^. In general, neurosurgeons consider RSW to be more common than SIADH in neurosurgical patients, whereas internists consistently regard RSW as a rare clinical entity. As reviewed elsewhere, RSW has in fact been found to be much more common than SIADH in neurosurgical patients and our reports of RSW occurring in patients without clinical cerebral disease introduce new challenges to determine the true prevalence of RSW ^5, 6^. This diagnostic dilemma poses a therapeutic dilemma that has significant clinical outcomes, such as the risk inherent in fluid restricting a volume-depleted patient with RSW. In addition, the recent awareness that even mild hyponatremia induces symptoms with potentially serious consequences including attention deficits, mental confusion, unsteadiness, and heightened risk for falls and fractures ^7– 9^, underscores the urgency needed to resolve this diagnostic dilemma so that the proper therapy can be instituted on first encounter with the patient ^7– 9^. Because of the erroneous perception that RSW is rare in neurosurgical patients and the unknown prevalence of RSW in patients without cerebral disease, the inappropriate fluid restriction or use of vasopressin receptor blockers in patients with RSW for an erroneous diagnosis of SIADH is probably much more common than what has been documented in the literature. Indeed, inappropriate fluid restriction of volume-depleted patients with RSW has been reported to have higher morbidity and mortality rates ^10– 12^. Our recent proposal to replace the inappropriate and outdated term, cerebral salt wasting, with RSW, therefore, has great clinical relevance because RSW would not in the past be considered unless the patient had evidence of cerebral disease ^1^.

We have utilized the persistence of an increased fractional excretion (FE) of urate after spontaneous or therapeutic correction of hyponatremia in RSW to differentiate it from SIADH where FEurate normalizes after correction of hyponatremia ^2, 12– 14^. This relationship between FEurate and serum sodium has been corroborated in several publications ^2, 10– 18^. The coexistence of an elevated FEurate with normonatremia appears to also identify patients with RSW without the need to correct a pre-existing hyponatremia. This notion was supported by earlier studies, which demonstrated natriuretic activity when the plasma of largely normonatremic neurosurgical and Alzheimer's disease patients with elevated FEurate were administered to rats ^19, 20^. In those rat studies, natriuretic activity was noted when the plasma was administered simultaneously by IV infusion and injection into the peritoneum, suggesting that some of the natriuretic factor may have entered the rats’ circulation by the latter route. We entertained the possibility then that the factor could be a protein small enough to be absorbed from the peritoneum, and if so, small enough to be filtered by the glomerulus as well. We postulated that the circulating protein would be filtered at the glomerulus, possibly saturating receptor sites in the renal tubule and thereby making its way into the urine. The present studies were therefore designed to investigate the presence of natriuretic activity in ammonium sulfate precipitates of urine from patients admitted predominantly to our neuroscience intensive care unit with normal or increased FEurate with or without hyponatremia in addition to SIADH.

## Materials and methods

### Clinical studies

Patients included in this report were recruited mainly from the neuroscience intensive care unit of Winthrop-University Hospital. The protocol was approved by the Institutional Review Boards for human research at Winthrop-University Hospital and the New York College of Osteopathic Medicine (recently renamed the New York Institute of Technology College of Osteopathic Medicine) where the in vitro sodium transport studies were performed. Signed consent was obtained for each patient before initiating the study.

The clinical portion of the study consisted of the inclusion of tests for serum uric acid and urine osmolality, and determination of sodium, potassium, uric acid, and phosphate levels in addition to the routine tests for each patient. Exluded from the study were patients with serum creatinine greater than 1.1 mg/dl, those with edematous states, pregnant females, and those with abnormal thyroid or adrenal function. All urine passed was kept on ice at the bedside, with the addition of 80% by volume ammonium sulfate, before being transferred to the refrigerator and laboratory.

### Cell culture

LLC-PK1 cells, an epithelial line derived from porcine proximal tubules, were obtained from the American Type Culture Collection (ATCC Manassas, VA, USA). Cells were maintained in Dulbecco’s modified Eagle’s medium (DMEM, Invitrogen) supplemented with 10% (v/v) fetal bovine serum (FBS, Invitrogen), 100 U/ml penicillin-100 µg/ml streptomycin (Invitrogen), 2 mM L-glutamine (Invitrogen) and 7.5% (w/v) sodium bicarbonate (Invitrogen). Cultures were grown at 37°C in a 5% CO _2_ atmosphere. Subculturing was performed every 3–4 days by trypsinization and reseeding at 1:20 dilution, and complete medium was replaced every 2 days. For experiments, cells were seeded on permeable supports that exposed both mucosal and serosal surfaces to the bathing media (12-well transwell filter inserts; Becton Dickenson Biocoat Control Inserts, 3 micron pore size, #354573) at a density of ≤ 2.5 × 10 ^5^ cells/well and grown to confluence. All cells were serum-starved at least 24 hours prior to experiment.

### Preparation of urinary natriuretic factor

Urine samples were collected from normal, SIADH, and salt-wasting patients. The natriuretic factor(s) was precipitated out with ammonium sulfate (80% w/v). The precipitate was dialyzed for 48 hours, 20x the volume with > 3 changes, using a 10kDa membrane against deionized water (dH _2_O) to remove the ammonium sulfate, and the sample was then lyophilized and reconstituted in dH _2_O and stored at -80°C until use. The precipitate was quantitated on the basis of its protein content by the Bradford method ^31^ and concentrations in the in vitro experiments were based on the protein concentration.

### Transport assay

At the start of each experiment, the culture medium of the LLC-PK1 cells was replaced by a transport medium composed of NaCl 140 mM, KCl 5 mM, CaCl _2_ 2 mM, MgCl _2_ 1 mM, HEPES buffer 15 mM, and L-glutamine 2 mM, pH 7.4. In some experiments, 5 mM glucose was also included. Calcein, a fluorescent derivative of fluorescein, was used to evaluate transepithelial passive leak rates across cultures. Calcein at a concentration of 16 micromolar was added to the serosal (lower) compartment of each well (Becton Dickenson, Falcon 12-well plates, #353503; 1 cm wells) and timed samples were taken from the mucosal side. Fluorescence of the samples was read at 495/515 nm excitation/emission wavelengths in a Turner Model 430 Spectrofluorometer, calibrated against graded concentration standards over the range 0–0.4 micromolar, and leak-down rates per hour calculated. To measure sodium transport, ^22^Na (Perkin Elmer) was diluted in serum-free DMEM/F12 (Invitrogen/GIBCO, cat. #11330) and added to the mucosal side of the transwells (final specific activity 1 to 6 microCi/mmol Na; 0.1–0.6 µCi/ml). Urinary precipitates (typically 5–20 µl/ml in dH _2_O) when applied were resuspended and introduced to the media bathing both the mucosal and serosal surfaces of the epithelial cultures. Sodium transport was measured based on liquid scintillation counting (Packard PE Ultima Gold Liquid Scintillation Cocktail, Cat. #6013329, in a Packard Tri-Carb 2900 LS counter) of aliquots taken from the serosal side at specified time points. Aliquots of 10 microliters were taken from each 2 ml serosal compartment every 30–60 minutes for 1–2 hours prior to sample addition (baseline) and for 3–4 hours after (experimental). Preliminary experiments indicated that a 30 to 60 minute period was sufficient to achieve an isotopic steady-state. The steady-state rate of transport under any condition was determined as the slope (change in cpm/time) of the linear regression line of the individual cold-side activity determinations (cpm/10 µl) versus time. Each individual patient was considered n = 1. Statistical significance was determined based on Students’ t-test with p = 0.05 or less being considered significant.

### Calculation of effect on Na movement

The effect of any treatment on sodium transport was determined as follows. After the initial baseline-establishing period, resuspended urinary precipitate was added to the desired concentration of protein in the mucosal and serosal bathing solutions of the appropriate culture wells. (In some experiments bovine serum albumin (BSA) as a control rather than urinary precipitates was added. The BSA (Sigma Chemical #A7030) was made as a stock solution of 1 mg/ml in deionized water and added to final experimental concentrations of 2.5, 5, or 10 micrograms/ml in the mucosal and serosal bathing solutions.) Equal volumes of Vehicle only (dH _2_O) were added at the same time to Vehicle wells run side-by-side in each culture plate. Each experimental condition (RSW patient urine precipitate, non-RSW Control patient precipitate, BSA, or Vehicle) was run in quadruplicate wells in a given experiment.

The effect of any experimental addition on sodium transport was determined by comparison of the baseline slope to the post-addition slope (delta-m, change in slope, = (slope after treatment) - (slope before)). The specific effect of urine precipitate or BSA on sodium transport in a given experiment was calculated versus any effect seen with vehicle in the same experiment, i.e. as the deviation of the slope change for Experimental wells (precipitate or BSA) from that for Vehicle-only wells, the deviation being given by the expression [(Δm _E_/Δm _V_) – 1] × 100%, where Δm _E_ is the slope change for Experimental wells caused by precipitate (or BSA) addition and Δm _V_ is that for wells to which only vehicle was added.

The effects of urine precipitates from RSW patients and from unaffected (non-RSW) Control patients was determined in separate experimental runs, each versus Vehicle-only wells run side-by-side in each experiment.

Finally, the specific effect of urine from the affected group of RSW patients was calculated versus any effect seen with the group of Control patient urines, i.e. as the mean effect for RSW/Experimental urine precipitates versus the mean effect for Control-patient urine precipitates.

The effect of RSW or Control urine precipitates on sodium movement was alternatively calculated as the slope after addition of precipitates versus the slope before addition in the same wells (without reference to Vehicle wells). However, that calculation proved to yield a higher degree of statistical scatter and was not ultimately used.

### Statistical analysis

Data are reported as the means ± S.E.M for both the number of patients and the number of experimental runs. Within a given run, data points are the mean values from four individual culture wells. Statistical differences were calculated based on the number of patients and were determined by unpaired Student's t test or analysis of variance where appropriate using Sigma Plot 9. Values of p < 0.05 were considered statistically significant.

## Results

### Patient characteristics

The study population was composed of patients with neurosurgical diseases of various etiologies who were admitted to the Winthrop-University Hospital neurosurgical intensive care unit, Table 1. After obtaining a signed informed consent, patients recruited into the study were divided into three groups, based on whether or not the patient had a normal or increased FEurate (normal 5–10%) or SIADH. Group I, controls, consisted of those with normal FEurate. Group II, SIADH, had increased FEurate, hyponatremia, and decreased baseline plasma renin and aldosterone levels. Group III, RSW, had increased FEurate with normonatremia, a combination that is consistent with RSW but not SIADH. Table 1 summarizes the pertinent clinical information, including diagnoses, age, gender, serum electrolytes, creatinine, phosphate and urate, urine sodium, and fractional excretion (FE) of sodium, urate and phosphate.

**Table 1.** Clinical features of patients in the study.

Group	Age Yrs	Gender	Na mmol/L	K mmol/L	Cr mg/dl	Urate mg/dl	PO _4_ mmol/L	UNa mmol/L	FENa %	FEurate %	FEPO _4_ %	Clinical diagnosis
I	72	M	141	4.2	0.6	6.2	3.7	43	0.12	4.02	3.73	Lumbar stenosis with laminectomy
	85	F	141	4.1	0.9	5.6	2.6	71	0.81	7.0	14.1	CVA, HTN
II	82	F	126	4.2	0.4	2.1	2.2	48	0.14	12.1	25.8	Poorly differentiated carcinoma of lung-metastatic to bone
	45	F	130	4.1	0.4	0.6	3.8	165	1.33	20.9	11.4	Congenital hydrocephalus-ductal stenosis of ventricle
III	83	F	146	3.7	0.7	2.6	2.7	140	1.0	48.4	13.9	Cerebral embolism with infarction, HTN
	83	M	140	4.1	1.0	3.4	3.4	130	3.4	13.9	18.0	SAH, right frontal infarct
	49	M	135	3.3	1.7	3.8	2.6	53	0.54	12.5	–	Renal Cell Ca met to 4 ^th^ ventricle
	50	F	141	3.6	0.6	1.6	2.7	147	1.71	13.2	7.73	Cerebellar AVM with hemorrhage
	84	M	141	4.1	1.0	3.2	3.5	114	0.96	12.3	12.9	Basal ganglia hemorrhage-intraventricular extension

### Transcellular transport of ^22^Na

**Figure 1.**
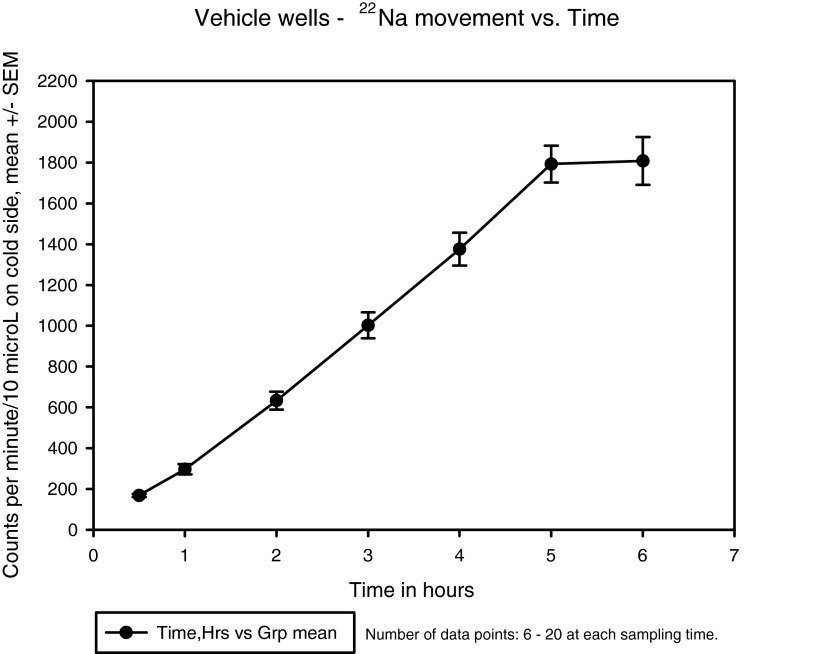
Transepithelial movement of ^22^Na across cultures with vehicle only added to the medium. Values at each time point are means ± SEM for 6 consecutive experiments with 3 or 4 wells per experiment at each time point. Number of data points at each time point are: 0.5 h (13); 1 h (19); 2 h (19); 3 h (20); 4 h (20); 5 h (20); 6 h (6).

Initial experiments revealed that certain culture conditions had to be met to achieve consistency of transport pattern from day to day. In order to achieve this consistency, the cell cultures had to go through a minimum of 10–12 passages after initial growth from frozen stocks, be plated at initial concentrations of 2.5 × 10 ^5^ per 2.3 cm diameter well [6 × 10 ^4^/cm ^2^] or less, and be utilized for transport studies 12–16 days after plating. The cultures were tested for confluence and transepithelial leakage using the fluorescent marker calcein, as described in the Methods. Using this method, cultures were shown to have passive leakage rates of < 1% per hour (0.7 ± 0.7%/hr, mean ± SD, range 0–2.2%/hr; n = 17 plates of 12 wells each). The cell monolayers also showed a high degree of restriction to movement of sodium isotope over a period of hours, indicating complete confluence and consistent formation of intercellular tight junctions throughout the study, as evidenced by the results of six consecutive experiments in which vehicle was the only addition to the bathing medium, shown in Figure 1. The results indicate that there was isotopic equilibrium within 30 minutes and consistent linear transport over 5 hours that was reproducible from day to day (Figure 1). Transcellular transport became erratic and nonlinear after 5 hours, thereby limiting all subsequent experiments to < 5 hrs.

**Figure 2.**
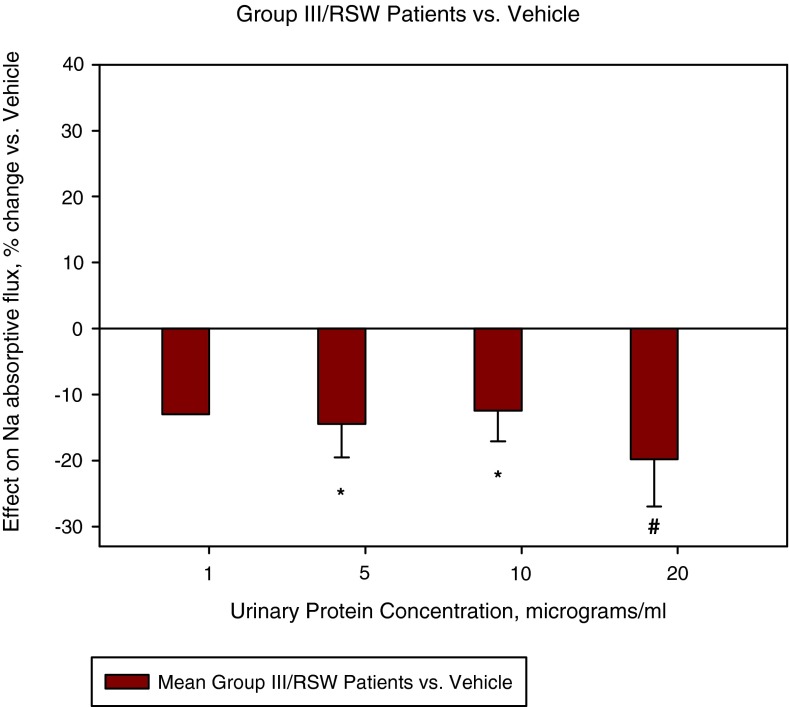
Effect of urine precipitates from Group III/RSW patients on transepithelial sodium movement, *versus vehicle*. Data are from 5 of 6 RSW patients with 6–16 experiments at each of the protein concentrations 5, 10, and 20 µg/ml. Values are versus vehicle set = 0 at each concentration. Significance levels vs. vehicle: At 5 µg protein/ml, P = 0.03 (*); 10 µg, P = 0.03 (*); 20 µg, P = 0.05 (#).

***Transepithelial movement of ^22^Na in cells exposed to precipitates from patient urine.*** Because data from Groups I and II were indistinguishable from each other, the data from both groups were pooled as a non-RSW control group. After attaining suitable culture conditions, resuspended precipitates from urine of Group III patients, applied to both mucosal and serosal bathing media, significantly inhibited sodium fluxes as compared to vehicle alone, outcomes that were distinctly different from those for Groups I and II. Of 6 patients initially in Group III, precipitates from 5 patients (Table 1) showed a decrease in sodium transport of 10–25% at concentrations greater than 3 µg protein/ml, as compared to vehicle alone in wells run side-by-side in each experiment, Figure 2 (P < 0.05 vs. vehicle).

**Figure 3.**
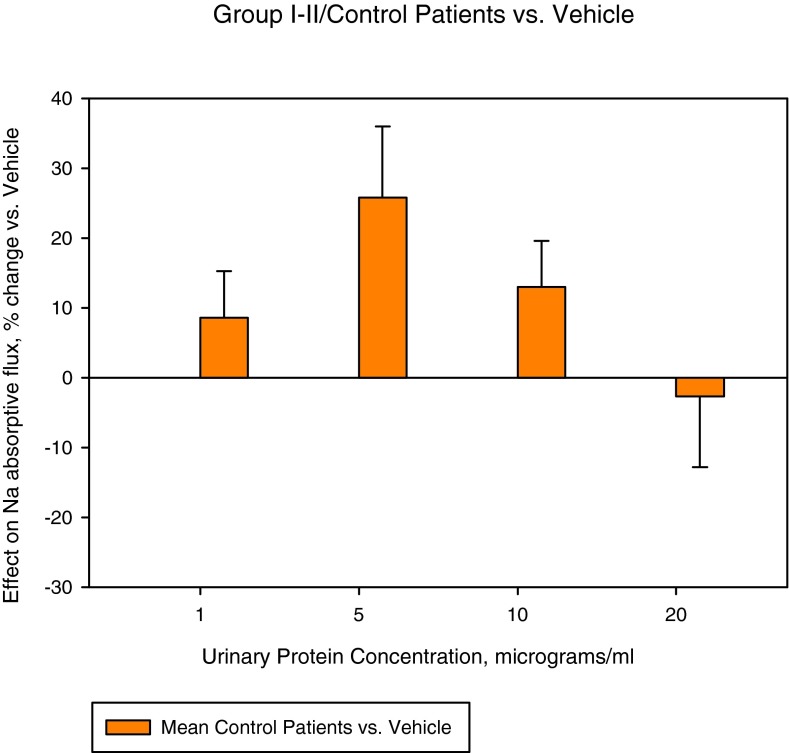
Effect of urine precipitates from Group I-II/Control patients on sodium movement across LLC-PK1 cultures, *versus vehicle*. Data from 4 non-RSW control patients (2 neurosurgical patients with normal FEurate and normonatremia and 2 SIADH) with 4–10 experiments at each protein concentration. Values are versus vehicle set = 0 at each concentration. Significance levels vs. vehicle: At 1 µg, protein/ml, P = 0.42; 5 µg, P = 0.09; 10 µg, P = 0.21; 20 µg, P = 0.82.

In contrast, urinary precipitates from 4 out of the 5 patients in Groups I/II (Table 1) did not inhibit sodium transport versus vehicle. In fact, mean sodium transport for Group I/II were mostly positive or increased, although statistical significance versus vehicle was not reached at any concentration in the ranges tested, Figure 3 (P = 0.09 to 0.82 @ 1–20 µg).

**Figure 4.**
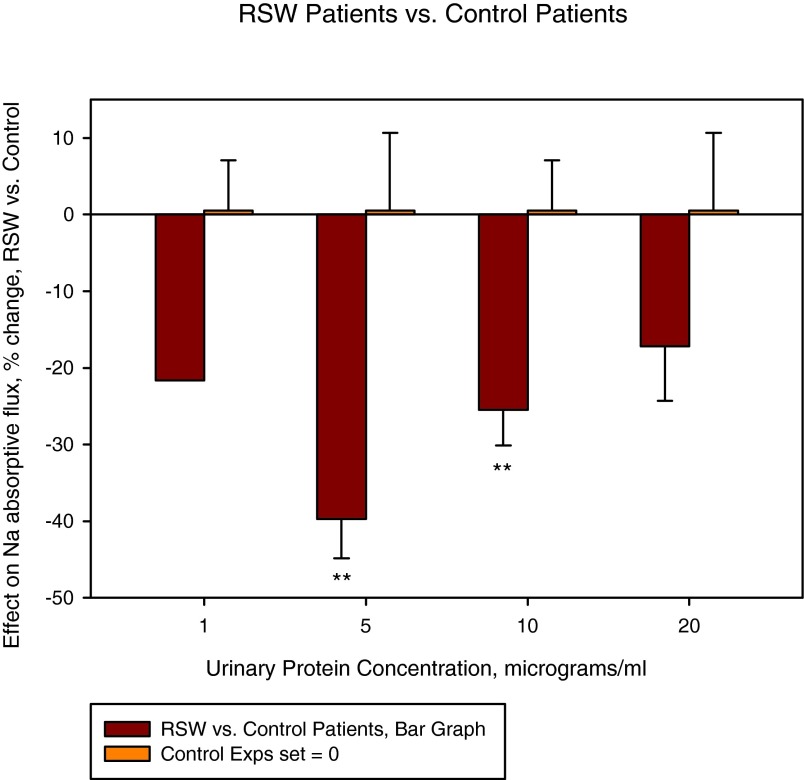
Effect of precipitates from the Group III/RSW patients on transepithelial sodium movement, *versus Group I-II/Control*. Values are versus Controls set = 0 at each concentration. Significance levels, Group III/RSW vs. Group I-II/Control: At 5 µg protein/ml, P = 0.012 (**); 10 µg, P = 0.013 (**); 20 µg, P = 0.24.

When the results for Group III patients were compared directly with Group I/II control patients, the differences were striking, as shown in Figure 4 and Table 2. Sodium transport by the Group III urine precipitates decreased significantly by 15–40% compared to Group I/II (P < 0.02 at 5 and 10 µg/ml).

**Table 2.** Effects of urine precipitates on sodium transport.

	Group I-II % change Vs. Vehicle ^(a)^mean ± SEM	Group III % change Vs. Vehicle ^(b)^mean ± SEM	Group III Vs. Group I-II mean ± SEM
1 µg/ml protein	+9 ± 7	-13	-22
5 µg/ml protein	+26 ± 10	-14 ± 5	-40 ± 5
10 µg/ml protein	+13 ± 7	-13 ± 5	-26 ± 5
20 µg/ml protein	-3 ± 10	-20 ± 7	-17 ± 7

Effect on absorptive sodium flux of urine precipitates from Group I-II vs. vehicle; Group III/RSW vs. vehicle; and Group III vs. Group I-II.

(a), Group I-II, 4 patients except for 1 µg/ml point (2 patients, 5 experimental runs).

(b), Group III, 5 patients except for 1 µg/ml point (1 patient, 3 experimental runs).

### Dose Response Studies

**Figure 5.**
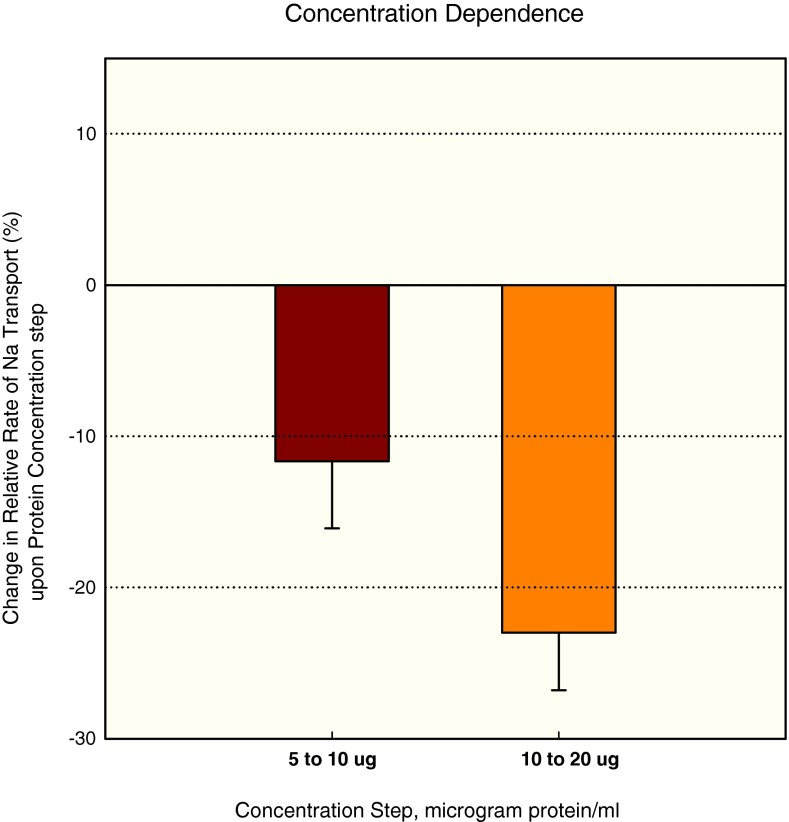
Effect of raising concentration of Group III/RSW urine precipitates: paired experiments (different concentrations in same experiment). Within a given experimental run, the effect of 10 µg protein was compared to that of 5 µg (each measured vs. vehicle) or the effect of 20 µg was compared to that of 10 µg (each versus vehicle). Significance levels: 10 µg vs. 5 µg, P = 0.046 (3 patients/6 experimental runs); 20 vs. 10 µg protein, P = 0.0038 (3 patients/5 experimental runs); 10 to 20 µg step versus 5 to 10 µg step, P = 0.045 (group comparison). Control patients showed no significant effect for either 5 to 10 or 10 to 20 µg concentration step (P > 0.3). P values calculated based on number of patients.

***Transepithelial transport of ^22^Na determined at different concentrations of precipitate on the same day for each patient in Group III.*** In these experiments, Group III precipitates consistently inhibited ^22^Na transport in a dose-dependent manner at concentrations of 5 to 10 µg protein and 10 to 20 µg (Figure 5). We also tested bovine serum albumin (BSA) at similar concentrations to assess whether a non-specific protein effect might be occurring. BSA did not significantly alter sodium transport from a baseline of zero (P = 0.2 to > 0.95 at concentrations of 2 to 10 µg/ml, data not shown). Therefore, the data indicate that a natriuretic factor(s) exists in the urine of Group III patients that inhibits transcellular sodium transport in cultured LLC-PK1 cells in a dose-dependent manner. These data are also consistent with previous demonstration of a dominant effect of plasma from neurosurgical and Alzheimer disease patients on rat proximal tubule transport of sodium and lithium ^19, 20^.

Tests for nonspecific protein effect by exposure to bovine serum albumin (BSA) This dataset contains the data and calculations for 7 control experiments which tested for a nonspecific protein effect by measuring the effects on sodium transepithelial movement of exposing cultures to bovine serum albumin (BSA), rather than urinary precipitates. The 'Summary' sheet summarizes the results of the experiments. The other 7 sheets (named by date of experiment) contain the raw data and calculations for the 7 experiments. The effect on sodium transport was calculated in the same way as in the experiments utilizing urinary precipitates.

Passive leakage across monolayers using calcein as a fluorescent marker This dataset tabulates data on passive leakage across the monolayers using a fluorescent marker, calcein, a derivative of fluorescein. The purpose was to gauge the degree of confluence and whether tight junctions had formed between the epithelial cells.
The sheet 'Leak rates' tabulates a summary of the data of the 17 calcein experiments in the raw data sheet. The sheet 'Raw data' contains the data and calculations of the 17 experiments.

Transepithelial sodium transport in neurosurgical patients with renal salt wasting This dataset contains the results of experiments measuring the transport of sodium across the monolayers. 22Na was used to make these measurements. The experiments tested the effects of resuspended urinary precipitates from renal salt wasting (RSW) patients and non-RSW Control patients. The amount of protein in the precipitates was used to quantitate the precipitates.
The 'Summary' sheet tabulates the results of the individual experiments described below. The heading numbers such as 5ug or 10ug refer to the concentrations of resuspended precipitates (in micrograms protein/ml) to which the cultures were exposed. In all experiments, cultures were exposed to precipitates on both the mucosal and serosal sides. Numbers tabulated are the effects of various concentrations of precipitate on transepithelial Na movement, expressed as percentages relative to that seen side-by-side in vehicle-only wells.
The control and RSW sheets contain the raw data and calculations, for 47 individual experiments in which the effects of precipitates from the urine of RSW-afflicted patients or non-RSW Control patients on sodium transport across cultured LLC monolayers were measured. Effects of the precipitates on sodium movements were calculated as described in the Methods section of the paper, in brief, the effect of treatment (change in the slope of the linear regression line of sodium movement after treatment versus before) in experimental culture wells versus the effect of vehicle treatment (change in the regression line after versus before treatment) seen in side-by-side Vehicle wells. The results for individual patients are grouped in sub-folders with the patients identified by index numbers.
Other numbers quoted are percent change in transepithelial sodium transport (caused by addition of urinary precipitate, indexed by its protein content) relative to vehicle addition, as described in Methods in body of the paper. (Briefly, relative to Na movement in side-by-side culture wells in which only vehicle was added to the transport medium).

## Discussion

This study demonstrates the presence of a factor, in the urine of 5 of 6 neurosurgical patients with increased FEurate and normonatremia, that inhibits transcellular transport of ^22^Na in LLC-PK1 cells grown to confluency in transwells (Group III). By contrast, ammonium sulfate precipitates of urine from neurosurgical patients with normal FEurate and normonatremia and those with SIADH did not inhibit transcellular transport of ^22^Na, suggesting that the natriuretic factor was present only in the urine of patients with increased FEurate and normonatremia. These data are consistent with previous rat clearance studies, which demonstrated natriuretic activity in the plasma of neurosurgical and Alzheimer’s disease patients who had increased FEurate and normonatremia as compared to two control groups, composed of (1) age- and gender-matched controls and (2) patients with multi-infarct dementia, both groups with normal FEurate and normonatremia ^19, 20^. These studies collectively support our proposal that an increased FEurate with normonatremia is consistent with RSW and represent an initial step to utilize the natriuretic factor as a biomarker for RSW ^1, 2^.

The plasma natriuretic factor in those previous rat clearance studies significantly increased FElithium and FENa without change in glomerular filtration rate, blood pressure, or urine flow rate. FElithium increased from control means of 24.0% and 27.2% to 36.6% and 41.7%, respectively, when plasma from neurosurgical and Alzheimer disease patients were injected into rats, and FENa changed from controls of 0.29% and 0.33% to 0.59% and 0.63% respectively ^19, 20^. Since lithium, in the absence of nonreabsorbable solutes such as mannitol, is transported over sodium pathways in the proximal tubule, the increases in FElithium suggested that the natriuretic factor had its major effect in the proximal tubule ^21, 22^. These data support our clinical impression that the increase in FEurate, a solute transported exclusively in the proximal tubule, indicated both a defect in solute transport in the proximal tubule of these patients and a defect involving more than one solute ^14^.

In those rat clearance studies, we administered 0.5 ml of plasma intraperitoneally 90 minutes prior to anesthetizing the animal and, under anesthesia, we rapidly infused 0.2 ml plasma as a primer and infused 1.8 ml of plasma at a rate of 0.01 ml/min over the next 3 hours. This maneuver was intended to increase the time of exposure of the natriuretic factor while minimizing duration of anesthesia to assure physiological integrity of the animal. FElithium and FENa increased progressively, attaining significance approximately 4 hours after intraperitoneal injection of plasma ^19, 20^. The fact that some of the human plasma was administered peritoneally raised the possibility that some of the natriuretic factor may have entered the rats’ circulation via that route. We reasoned that the natriuretic factor could be a protein of small enough size to pass through the peritoneal membrane, and hence small enough to be filtered at the glomerulus, possibly saturate downstream tubular receptor sites, and be excreted in the urine. In the present study then, we therefore collected urine from prospective study patients and tested ammonium sulfate precipitates of urine for natriuretic activity in a transwell culture system. The inhibition of transcellular sodium transport by urinary ammonium sulfate precipitates of Group III patients suggests that the kidneys had indeed excreted the natriuretic factor and perhaps concentrated it as well. Clearly, the combination of small sample-sizes required by our *in-vitro* bioassay, together with a ready experimental source of the natriuretic factor in urine, should simplify purification and identification of the natriuretic factor going forward.

Atrial/brain natriuretic peptides (A/BNP) have been suggested as a possible cause of RSW. A/BNP has in fact been shown to be increased in conditions often *associated* with RSW such as subarachnoid hemorrhage, as well as in non-salt-wasting conditions such as SIADH and in salt-retaining conditions such as congestive heart failure ^27– 29^. However, the precipitates employed in the in vitro sodium transport experiments reported here were exhaustively dialyzed against 10 kDa-cutoff membranes. Hence, it’s unlikely that ANP (human MW_3080) or BNP (human MW_3464) were retained and present to any appreciable extent in the retentate to which the cultured cells were exposed. Furthermore, the physiological effects of ANP and BNP are distinctly different from those of the natriuretic factor observed in the earlier rat clearance studies ^23– 26^. A/BNP increase GFR, decrease renal blood flow and blood pressure, and increase urine flow rate and sodium excretion but have a greater decreasing effect on distal as compared to proximal tubule sodium transport. The natriuretic factor in our previous rat clearance studies did not increase GFR, decrease blood pressure or increase urine flow rate, and the increase in FElithium far exceeded the increase in FENa, suggesting that the natriuretic factor had a greater effect on proximal than distal tubule sodium transport ^19, 20^, concordant with the other considerations discussed above which indicate defects in proximal tubule solute transport in RSW. The properties then of the natriuretic effect noted in the plasma of patients in the earlier rat study and urine of patients in the present study with a very high probability for RSW strengthens our proposal that A/BNP is an unlikely contributor to RSW.

It should also be noted that the predominant stimuli for increased A/BNP release are related to increases in extracellular volume and blood pressure, the opposite of what is seen in RSW ^23, 24^. This was seen strikingly in the low normal value of 35 pg/ml of ANP we reported in a volume-depleted patient with unequivocal RSW ^6^, and does not support the common perception that A/BNP is the major natriuretic factor in RSW. This instructive case of RSW without cerebral disease had a decreased blood volume of 7.1% as determined by the gold standard radioisotope dilution method, using 51Cr-tagged red blood cells and 131I-tagged albumin, and increased baseline plasma renin and aldosterone levels, features all consistent with RSW. The subsequent infusion of saline removed the stimulus for ADH secretion and permitted the coexisting hypo-osmolality to inhibit ADH secretion, increase free water excretion and result in prompt correction of hyponatremia within 48 hours after initiation of saline therapy ^6^, responses again consistent with RSW.

Because of divergent therapeutic goals for SIADH and RSW (fluid restriction for SIADH and fluid supplementation for RSW), it is important to differentiate SIADH from RSW and thus avoid selecting the incorrect mode of therapy that can lead to increased morbidity and mortality. The patient with an increased FEurate with normonatremia without going through a phase of hyponatremia probably has RSW and should be treated with saline. In the case of the patient who presents with hyponatremia, there are ample clinical data to support the notion that the persistence of an elevated FEurate in RSW can be contrasted to normalization in SIADH after correction of the hyponatremia. However, this unique association between FEurate and hyponatremia is limited by the need to correct a preexisting hyponatremia, which is not always present, as just noted, and in any case may be difficult to achieve on a timely basis, and is hence not ascertainable on first encounter with the patient when therapeutic decisions are being made. On the other hand, a normal FEurate in a nonedematous hyponatremic patient consistently identifies patients with a reset osmostat, who fall into a different physiological and treatment group than the patients being addressed in this communication ^30^. The unique relationship between serum sodium and FEurate can, therefore, be utilized to establish or rule out the diagnosis of a reset osmostat and to differentiate RSW from SIADH, but for practical reasons can not be applied in every case ^2^. Diagnostic dilemmas that remain could be resolved simply if the natriuretic factor in the current study can be identified and validated as a biomarker of RSW. We believe the data from the current study are a major advancement to that ultimate identification of the natriuretic factor and developing the factor as a potential biomarker of RSW.
