# Supplementary material for: Demonstration of natriuretic activity in urine of neurosurgical patients with renal salt wasting
Source: F1000Res. 2013 Jun 13;2:126. Originally published 2013 May 10. [Version 2] doi: 10.12688/f1000research.2-126.v2 (PMC3752684; doi:10.12688/f1000research.2-126.v2)
Supplement: Peer review history

# Peer review: Referee response for version 2

**DOI:** 10.5256/f1000research.1689.r1737

**Published:** 2013-09-06

**Authors:** Pravin C Singhal

**Affiliations:** 1 Division of Kidney Diseases and Hypertension, Hofstra North Shore LIJ Medical School, Manhasset, NY, USA

I have read this submission. I believe that I have an appropriate level of expertise to confirm that it is of an acceptable scientific standard.

# Peer review: Referee response for version 2

**DOI:** 10.5256/f1000research.1689.r1030

**Published:** 2013-06-26

**Authors:** Albert Dreisbach

**Affiliations:** 1 Department of Medicine, University of Mississippi Health Care, Jackson, MS, USA

The article takes into account the suggested revisions and have improved the article.

I have read this submission. I believe that I have an appropriate level of expertise to confirm that it is of an acceptable scientific standard.

# Peer review: Referee response for version 1

**DOI:** 10.5256/f1000research.858.r956

**Published:** 2013-05-20

**Authors:** Albert Dreisbach

**Affiliations:** 1 Department of Medicine, University of Mississippi Health Care, Jackson, MS, USA

This a well designed study of a potentially significant and novel natriuretic factor in the urine of patients with elevated fractional urate excretion with renal salt wasting. This factor appears to have properties that distinguish it from ANP and BNP and may be entirely unique.

Minor criticisms:

- Methodological descriptions seem to run into the results and discussion which maybe more appropriate in the methods section. For example the first paragraph under the “Transcellular Transport of ^22^Na” in the results section describes the calibration of system might be more appropriate under methods.
- If any objective blood volume data available  it would support the differentiation of the RSW and SIADH patients.
- The use Uosm instead of meq/L or mmole/ L for  Na and K is not the usual convention.

I have read this submission. I believe that I have an appropriate level of expertise to confirm that it is of an acceptable scientific standard.

# Peer review: Referee response for version 1

**DOI:** 10.5256/f1000research.858.r942

**Published:** 2013-05-13

**Authors:** Pravin C Singhal

**Affiliations:** 1 Division of Kidney Diseases and Hypertension, Hofstra North Shore LIJ Medical School, Manhasset, NY, USA

The authors investigated salt wasting properties in the urine of neurological patients with salt wasting. They have used *in vitro* techniques to achieve their outcome. These studies are novel though preliminary and worth publishing.

I have read this submission. I believe that I have an appropriate level of expertise to confirm that it is of an acceptable scientific standard.
